# The utility of clusters and a Hungarian clustering algorithm

**DOI:** 10.1371/journal.pone.0255174

**Published:** 2021-08-04

**Authors:** Alfred Kume, Stephen G. Walker

**Affiliations:** 1 Department of Mathematics, Statistics & Actuarial Science, University of Kent, Canterbury, Kent, United Kingdom; 2 Department of Mathematics and Department of Statistics & Data Science, University of Texas at Austin, Austin, Texas, United States of America; Utrecht University, NETHERLANDS

## Abstract

Implicit in the *k*–means algorithm is a way to assign a value, or utility, to a cluster of points. It works by taking the centroid of the points and the value of the cluster is the sum of distances from the centroid to each point in the cluster. The aim in this paper is to introduce an alternative way to assign a value to a cluster. Motivation is provided. Moreover, whereas the *k*–means algorithm does not have a natural way to determine *k* if it is unknown, we can use our method of evaluating a cluster to find good clusters in a sequential manner. The idea uses optimizations over permutations and clusters are set by the cyclic groups; generated by the Hungarian algorithm.

## 1 Introduction

Cluster analysis is one of the most important problems within the data sciences. Identifying groups of similarity from among a data set has multiple applications. The common approach to determining a cluster from a data set is via the minimization of an objective function once the number of clusters has been set a priori. A comprehensive review is provided in [1, 2], for example. Another recent review of clustering algorithms is given in [3] with an emphasis on comparisons between the competing approaches.

Generally, clustering methods fall into one of three classes:

1. *Model–based approaches*. Mixture models are used to model the data and with statistical estimation of the parameters of the model; which include the number of components in the mixture, the number of clusters alongside the density used to estimate each cluster are available.
2. *Partitioning–based approach*. Here a given number of clusters is pre–defined and the data are optimally partitioned into this number of groups via some objective function.
3. *Tree–based approach*. From a given cluster; either new clusters are formed from these (top down) or these clusters are tied together into a smaller set of clusters (bottom–up). It must be pointed out that there is no natural termination to this approach and hence the appropriate number of clusters remains elusive.

The algorithm we are proposing in this paper is tree–based, also known as hierarchical clustering, and bottom up, also known as *agglomerative* clustering. For a review, see, for example, [4].

Starting with as many clusters as data points, these algorithms merge clusters until all data elements belong to the same cluster. At each step the two clusters which are separated by the least distance are combined. The distance is known as the linkage function; for example

$$d\left(S_{j},S_{k}\right)=\underset{x\in S_{j},y\in S_{k}}{\text{min}}d\left(x,y\right)$$

where *d*(*x*, *y*) is the natural, usually Euclidean, distance between elements *x* and *y*, and *S* denotes a cluster. The tree–based approaches are popular due to the visual appeal of a dendogram. However, the problem after the tree is constructed, is to determine the best cluster; see [5].

However, rather than methodically combining nearest clusters to produce the tree, we use a new utility function, which we introduce later, to combine clusters. The idea is that the new clusters are represented by the cyclic groups of an optimal permutation between elements. This permutation is described later. The basic idea is that the value assigned to the cluster by the cycle permutation minimizes the walking distance to reach each element of the cluster once. This provides a natural stopping rule for the further combination of clusters.

To motivate our utility or value for a cluster, we discuss one of the most popular clustering approaches, *k*–means; see [6] for one of the original papers on this algorithm, which is the most common unsupervised learning algorithm in data science. More recent contributions to *k*–means include the paper [7] which modifies the traditional algorithm to merge any two clusters with centroids which are close to each other. To describe the algorithm; if *k*, the number of clusters is known, the aim is to minimize over non–empty sets (*S*_1_, …, *S*_*k*_), the union of which is {1, …, *n*}, the function

$$\begin{array}{c} O\left(S_{1},\ldots ,S_{k}\right)=\sum_{j=1}^{k}\sum_{i\in S_{j}}d\left(c_{j},x_{i}\right), \end{array}$$ (1)

where *c*_*j*_ is the centroid of points in *S*_*j*_ and *d* denotes a measure of distance between points; for example, the square of the Euclidean distance is the most common. The centroid *c*_*j*_ is generally provided by the Fréchet mean; i.e.

$$c_{j}=\arg\min_{c}\sum_{i\in S_{j}}d\left(c,x_{i}\right).$$

Of course, if the distance is Euclidean, as is commonly assumed in the literature, then the centroid could be the usual mean. Implicit in the *k*–means algorithm is the assignment of

$$\begin{array}{c} v\left(S\right)=\sum_{i\in S}d\left(c,x_{i}\right) \end{array}$$ (2)

as a utility value of a cluster *S* with centroid *c* and cluster members (*x*_*i*_)_*i* ∈ *S*_. In fact, as far as we have been able to ascertain, there is no competing idea for the valuation of a cluster. The basis of the present paper is an alternative valuation of a cluster; indeed, this is the main contribution of our paper. We introduce our new value for a cluster in section 2.

Hence, we adopt the basic idea of agglomerative clustering, but use a value assignment to a cluster to achieve this, which is different to that used in centroid approaches, such as *k*–means. Whereas the distance based approach to agglomerative clustering and *k*–means do not provide a general optimal cluster (a tree of clusters for the former and a fixed *k* for the latter), our hybrid approach provides an optimal cluster, as the tree has a natural stopping point.

The basic *k*–means algorithm needs refinement when *k* is unknown, and there is no recognized single procedure for determining *k* and a corresponding set of *k* clusters. Rather, a two–step process is implemented whereby first an optimal choice for *k* is found, which is non–trivial, and then *k*–means is implemented with this choice of *k*. The most popular approach is the *elbow* method; see [8]. This implements a number of *k*–means algorithms for a range of *k* and then selects that value for which the graph presents an elbow looking shape, based on the overall sum of squares of points. Alternative strategies include the gap statistic; [9].

Other approaches to clustering popular within the machine learning community are Bayesian mixture models; in particular, the mixture of normal model;

$$\begin{array}{c} p\left(x\right)=\sum_{l=1:k}w_{l\;k}\;\text{N}\left(x|\mu_{l},\sigma^{2}\right). \end{array}$$ (3)

Here *N* stands for the normal density function, the (*w*_*lk*_) are weights which sum to 1, the (*μ*_*l*_) are the location parameters and *σ*^2^ the common variance. Finally, *k* represents the number of clusters though each must be well represented by a normal density otherwise *k* will be overestimated. In fact, overestimation is a serious concern for this type of model. The model falls within *Bayesian nonparametric* methodology; see, for example, [10]. A nice paper looking at *k*–means from a Bayesian nonparametric perspective is the one by [11]; see also [12]. However, a problem with modeling the data is that a cluster is required to conform to a simple model, such as the normal. It is well known that estimating the number of clusters is difficult, based on the estimation of the parameters. See [13, 14].

Combinations of types of data are also problematic for establishing a centroid and therefore a procedure which works solely using distances between data points to value a cluster is attractive. Our valuing approach to a cluster avoids the use of a centroid. Given this motivation, in our paper, we introduce a sequential algorithm which provides clustering with an unknown number of clusters and which can be seen as a development of the *k*–means and elbow algorithms in that the number of clusters is non–increasing and converges as the iterations progress. Each iteration can reduce the number of clusters, while never increases, so there is guaranteed convergence to a fixed number. As the algorithm proceeds the record is kept of which points are within each cluster.

The remainder of the paper is as follows: in Section 2 we present our idea for assigning a value to a cluster, and in section 3 we describe the algorithm for obtaining the number and elements of the clusters. This involves an optimization routine over permutations within each iteration and relies on the Hungarian algorithm. In Section 4 we present a number of illustrations. When our algorithm matches the number of clusters from alternative algorithms with a chosen cluster size, such as *k*–means, the clusters are effectively the same. Hence, in Section 4.5 we focus on comparing our number of clusters with ideas based on common strategies including the “elbow”, “gap” and “silhouette” methods. Finally, Section 5 concludes with a discussion and some idea for future work.

## 2 Valuing a cluster

Our alternative to (2) for assigning a value to a cluster *S* is given by

$$\begin{array}{c} u\left(S\right)=\underset{\sigma \in A}{\text{min}}\sum_{i\in S}d\left(x_{i},x_{\sigma (i)}\right) \end{array}$$ (4)

where *A* is the set of permutations on *S* which have a single cycle. For example, if |*S*| = 3, then a *σ* ∈ *A* could be *σ*(1) = 2, *σ*(2) = 3 and *σ*(3) = 1. So *u*(*S*) is the shortest walk to reach each point in *S* once only; starting and ending at one of the points. We also see this as a natural value for a cluster. It is the minimum distance to journey to all the points once and return to the original starting point. To us it seems this is a more natural way to define a cluster, though as with all utilities, there is no available proof of any kind it is superior or optimal compared to (2).

**Fig 1:**
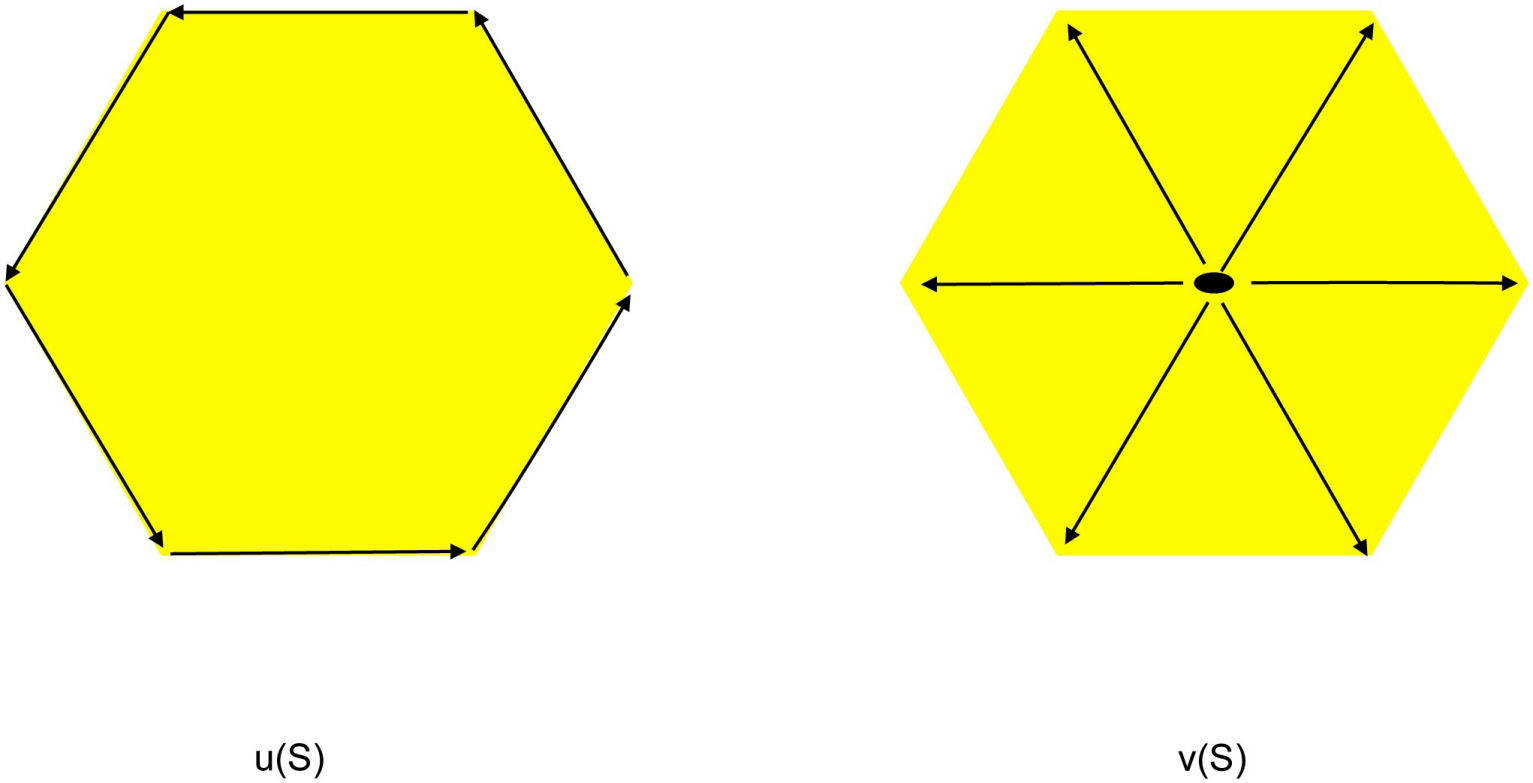
Left figure is *u*(*S*) and right figure is *v*(*S*).

The best we can do is highlight a case where the value (4) is natural and yet for the same case the value (2) is not. So here we compare the two metrics for evaluating the value of a cluster *S*. When |*S*| = 2, the values are the same; being the Euclidean distance between the two points. On the other hand, to see clearly the differences involved, consider a cluster involving a set of 6 points as the vertices of a symmetric hexagon. See Fig 1. The left figure describes the value of *u*(*S*) with arrows going round the perimeter of the hexagon; whereas the right figure describes the value of *v*(*S*), with the arrows emanating from the centroid to the vertices.

To provide some mild criticism of the *v*(*S*) in (2), consider now a cluster with points at the vertices of a perfect square with sides of length *a*. Then *u*(*S*) = 4*a* and $v\left(S\right)=2a\sqrt{2}$. Now suppose we move two of the points so that there are two points on each of the two diagonally opposite vertices. This will present a clearly different cluster than before where each point is at a vertex. Now $u\left(S\right)=2a\sqrt{2}$, a smaller value to before, as clearly the cluster has become more condensed. On the other hand, the new value for *v*(*S*) remains at $2a\sqrt{2}$; yet as we have mentioned, the two clusters are quite different. To see this point made with Fig 1, if we move all six points to one of two vertices opposite each other, the value of the cluster used by the *k*–means algorithm; i.e. *v*(*S*), stays the same. On the other hand, our value of the cluster *u*(*S*) is reduced, as it should be.

There are alternative ways to assign a utility to a cluster which avoids the notion of a centroid; a big advantage in general metric spaces, and one such recent idea appears in [15]. To describe this value of a cluster consider Ω = {*x*_1_, …, *x*_*n*_} to be a finite set of points, and let *S* be a proper subset of Ω. A value to cluster *S* is written as *γ*(*S*). The value is based on the metric *d* and the notion of the measure of cohesion between two points *x* and *y* in Ω defined to be

$$\gamma \left(x,y\right)=\frac{1}{n}\sum_{z\in \Omega }d\left(x,z\right)+\frac{1}{n}\sum_{z\in \Omega }d\left(y,z\right)-K/n^{2}-d\left(x,y\right),$$

where

$$K=\sum_{z_{1},z_{2}\in \Omega }d\left(z_{1},z_{2}\right).$$

It is interpreted as a “binding” force between two points and satisfies some key properties such as being symmetric, *γ*(*x*, *x*) ≥ 0, *γ*(*x*, *x*) ≥ max_*y*∈Ω_ *γ*(*x*, *y*). Then

$$\gamma \left(S\right)=\sum_{x,y\in S}\gamma \left(x,y\right).$$

If we define *d*(*A*, *B*) = ∑_*x*∈*A*,*y*∈*B*_ *d*(*x*, *y*) then

$$\gamma \left(S\right)=2\left(1-|S|/n\right)\left(|S|/n\right)\;d\left(S,S^{\prime }\right)-{\left(|S|/n\right)}^{2}d\left(S^{\prime },S^{\prime }\right)-{\left(1-|S|/n\right)}^{2}d\left(S,S\right).$$

That this is the explicit value assigned to a cluster follows from the objective function to be optimized for setting the clusters being

$$Q\left(S_{1},\ldots ,S_{K}\right)=\sum_{k=1}^{K}\gamma \left(S_{k}\right).$$

The concern about such utilities is that it is easy to confirm that *γ*(*S*′) = *γ*(*S*). Hence, if 3 clusters are sought, it is not 3 utilities which come into play; since if *S*_1_ and *S*_2_ are two clusters, with arbitrary utility, the third set *S*_3_ = Ω − *S*_1_ − *S*_2_ must have utility *γ*(*S*_1_ ∪ *S*_2_, *S*_1_ ∪ *S*_2_). This connection arises due to the value of a cluster ultimately depending on Ω which can be deemed as inappropriate.

## 3 The Hungarian clustering algorithm

In this section we provide an overview and then a detailed description of the proposed clustering algorithm which uses (4). To describe an iteration, suppose we start with *k* clusters $S\left(k\right)=\left(S_{1},\ldots ,S_{k}\right)$, having the *k* centroids $C\left(k\right)=\left(c_{1},\ldots ,c_{k}\right)$. Hence, *S*_*j*_ ∩ *S*_*l*_ = ∅ for *j* ≠ *l* and $\cup_{j=1}^{k}S_{j}=\left\{1,\ldots ,n\right\}$. The output of one iteration of the algorithm will be *k*′ ≤ *k* clusters, say $S^{\prime }\left(k^{\prime }\right)=\left(S_{1}^{\prime },\ldots ,S_{k^{\prime }}^{\prime }\right)$, with corresponding centroids $C^{\prime }\left(k^{\prime }\right)$, and where each $S_{j}^{\prime }$ is a union of known clusters from S(k).

To detail the algorithm, first define

$$\begin{array}{c} \eta =\frac{\sum_{j<l}d\left(c_{j},c_{l}\right)}{k(k-1)} \end{array}$$ (5)

to be half the average distance between the *k* centroids. This choice will be explained and motivated later. Then define the objective function to be minimized as

$$\begin{array}{c} O\left(\sigma \right)=\sum_{j=1}^{k}[d\left(c_{j},c_{\sigma (j)}\right)+\eta 1\left(\sigma \left(j\right)=j\right)], \end{array}$$ (6)

where the minimization is over all permutations *σ* on {1, …, *k*}. A detailed description of the optimization procedure is given in Section 3.

The optimal *σ*, say $\hat{\sigma }$, is a product of cycles of permutations. Thus, $\hat{\sigma }$ will have a number of cycles, or orbits, which are upper bounded by *k*, and denote this number of cycles by *k*′. Each cycle will form the new clusters; hence we obtain $S^{\prime }\left(k^{\prime }\right)$. The algorithm is repeated until *k*′ = *k*; i.e. $\hat{\sigma }$ is the identity permutation.

For example, if *k* = 6 and the clusters are denoted by sets *S*_1_, …, *S*_6_, and $\hat{\sigma }=\left(1\right)\left(5,2,3\right)\left(6,4\right)$, then *k*′ = 3, with the new clusters givenby $S_{1}^{\prime }=S_{1}$, $S_{2}^{\prime }=S_{2}\cup S_{3}\cup S_{5}$ and $S_{3}^{\prime }=S_{4}\cup S_{6}$.

The motivation for the algorithm is to cluster centroids by putting together those which are sufficiently close compared to the penalty term *η*. So, for example, if all inter–centroid distances are greater than *η* then the algorithm will stop. Hence, the penalty term is crucial.

### 3.1 Setting *η*

Our choice of *η* is motivated by the work of [15] who consider relative distances between points. Given points *S* = {*c*_1_, …, *c*_*k*_}, they consider the relative distance between points *c*_*i*_ and *c*_*j*_, and *j* ≠ *i*, as

$$RD(c_{i}\|c_{j})=d\left(c_{i},c_{j}\right)-k^{-1}\sum_{l\in S}d\left(c_{i},c_{l}\right).$$

The relative distance from a random point to a point *c*_*j*_ is defined as

$$R\left(c_{j}\right)=k^{-1}\sum_{l\in S}RD(c_{j}\|c_{l}).$$

The cohesion measure between points *c*_*i*_ and *c*_*j*_ is

$$\gamma \left(c_{i},c_{j}\right)=RD\left(c_{j}\right)-RD(c_{i}\|c_{j})$$

and satisfies a number of key conditions listed in Proposition 4 of [15]. The cohesion measures determines how suited *c*_*i*_ and *c*_*j*_ are to be in the same cluster. So *c*_*i*_ and *c*_*j*_ are cohesive if *γ*(*c*_*i*_, *c*_*j*_)≥0 which can be understood as a “binding force” between the points.

On the other hand, the self cohesion for point *c*_*j*_ is given by

$$\gamma \left(c_{j},c_{j}\right)=\frac{2}{k}\sum_{l=1}^{k}d\left(c_{j},c_{l}\right)-\frac{1}{k^{2}}\sum_{l,m=1}^{k}d\left(c_{l},c_{m}\right).$$

We take *η* to be an average of these self cohesions; to see exactly what average note that

$$\sum_{j=1}^{k}\gamma \left(c_{j},c_{j}\right)=k^{-1}\sum_{i=1}^{k}\sum_{j=1}^{k}d\left(c_{j},c_{i}\right).$$

Due to the symmetry of *d* we divide by 2, to avoid double counting, and we have a loss of one degree of freedom due to the relative notion of the measure; i.e. so we take the average as

$$\eta =\frac{1}{2(k-1)}\sum_{j=1}^{k}\gamma \left(c_{j},c_{j}\right)$$

which becomes

$$\eta =\frac{\sum_{i=1}^{k}\sum_{j=1}^{k}d\left(c_{i},c_{j}\right)}{2k(k-1)},$$

and which is (5). Clearly, the smaller the value, the better a cluster are the set of points.

This provides, unlike existing agglomerative clustering algorithms, an automatic stopping rule. A set of points will not be clustered into any further separate clusters when

$$\eta <\min_{\sigma \in C}\;\;\frac{1}{k}\sum_{i=1}^{k}d\left(c_{i},c_{\sigma (i)}\right)$$

where *C* is the set of permutations on {1, …, *k*} with a single cycle. That is, the average self cohesion is more attractive than the corresponding average of distances associated with any permutation with a single cycle.

### 3.2 Motivation for *O*(*σ*)

We provide a motivation by taking a new look at the *k*–means algorithm. Suppose we have *n* points, *x*_1:*n*_, and want to put them into *k* ≤ *n* categories. The *k*–means approach minimizes

$$O\left(S_{1},\ldots ,S_{k}\right)=\sum_{j=1}^{k}\sum_{i\in S_{j}}d\left(c_{j},x_{i}\right)=\sum_{j=1}^{k}v\left(S_{j}\right).$$

where $c_{j}=\sum_{i\in S_{j}}x_{i}/\left|S_{j}\right|.$ In other words, the “value” of a cluster *S* with centroid *c* is given by (2). The smaller the value for *v*, the better the cluster, and this choice of *v* is easy to understand.

On the other hand, we use a different value for a cluster; i.e. (4). However, while the *k*–means approach does not extend naturally to an optimization problem over *k*; our approach with values based on *u*(*S*) does. That is, now with *σ* a permutation on {1, …, *k*}, we want to minimize

$$l\left(S_{1},\ldots ,S_{k},k\right)=\sum_{j=1}^{k}\sum_{i\in S_{j}}d\left(x_{i},x_{\sigma_{j}\left(i\right)}\right)$$

where *σ* = (*σ*_1_, …, *σ*_*k*_) are the permutation cycles within *σ*. This will also yield a *k*.

In practice, as with the *k*–means algorithm, the algorithm behind minimizing *O*(*σ*); i.e. (6), is quite straightforward and implemented recursively. From *k* clusters, the next iteration provides a new set of clusters of size *k*′ ≤ *k*, via the cycles of $\hat{\sigma }$, which minimizes the total sum of traveling distance accumulated from the distances between the points in a cycle. In other words, we are minimizing over all *k*′ and $S^{\prime }$, $\sum_{j=1:k^{\prime }}D\left(S_{j}^{\prime }\right)$, where $D(S_{j}^{\prime })$ are the internal distances between the elements within $S_{j}^{\prime }$.

We understand this to be a perfect recursive notion for improving on a clustering to a smaller degree. Of course, this could result in *k*′ = *k* since the “distance” from a cycle with a single point is given by *η*; the explanation of which has just been provided. See Algorithm 1.

**Algorithm 1**: Sequential clustering

Set S(k) and C(k) and *N* = 0;

**while** *N* = 0 **do**

 Minimize $\;O\left(\sigma \right)=\sum_{j=1}^{k}\left[d\left(c_{j},c_{\sigma (j)}\right)+\eta \;1\left(\sigma \left(j\right)=j\right)\right]$;

 **if** *k*′ < *k* **then**

  Get $S^{\prime }\left(k^{\prime }\right)$ and $C^{\prime }\left(k^{\prime }\right)$ from $\hat{\sigma }$, S(k) and C(k);

 **else**

  *N* = 1

 **end**

**end**

### 3.3 Implementation: The Hungarian algorithm

The key to the implementation of the sequential clustering is the *Hungarian algorithm*; see [16]. The input consists of a *k* × *k* matrix with non–negative elements. Typically the algorithm it is used to solve an assignment problem; namely the rows consist of “workers” and the columns consist of “tasks”. The entry in the (*i*, *j*)th position would represent the amount in dollars the *i*th worker would charge on task *j*, written as *d*(*i*, *j*). Clearly, the objective for the manager would be to make the worker–task assignments to minimize costs and hence is looking for a permutation *σ* minimizing ∑_*i*=1:*n*_ *d*(*i*, *σ*(*i*)). There are *n*! possible permutations. However, the procedure it uses to find the optimal solution means it runs in order of time *n*^3^. For us the costs of assignments are distances and the optimal permutation yields, or can be decomposed, into permutation cycles, also known as “orbits”. See, for example, [17]. It is these orbits which form the new set of clusters.

Here, we describe the implementation of our algorithm in the clustering context for generating the mapping *σ*; i.e. for minimizing *O*(*σ*). Let us assume initially that we are given a *k* × *k* symmetric matrix of distances such that the diagonal distance entries are not zero but equal to some given value defined as *η*. We are then focusing on the assignment problem which addresses the optimal match which in our case is a permutation *σ* for a given set of components *k*. Note, we are not looking for the permutation of the type *σ* = *σ*^−1^ as required by [18] in a non–bipartite matching context; and see, for example, [19] for a comprehensive review of various uses of optimal matching in statistics.

Our optimisation problem can be solved with the aid of linear programming algorithms; in fact a particular version of such algorithms is the Hungarian method which finds the optimal solution in a polynomial time of order *O*(*k*^3^). Such an algorithm is carried out as a four step procedure as follows, with *M* being the distance matrix i.e. *M*_*i*,*j*_ = *d*(*i*, *j*):

This implies that the computation cost of our clustering iterations are manageable and as *k* deceases the convergence will be achieved with increased speed see [20]. In particular, our calculations are carried out in R (https://www.r-project.org/) and the key packages for performing the optimization for the cross matching is based on the function *Solve_LSAP* which implements the Hungarian algorithm. For more on the Hungarian algorithm, see Algorithm 2 and [21]. The overall algorithm proceeds as described in Algorithm 3.

**Algorithm 2**: Hungarian algorithm

**Step 1** Subtract the minimum value from each row of *M* (so in each row the mimimal value will be zero)

**Step 2** Subtract the minimum value from each column of *M* (so in each column the minimal value will be zero)

**Step 3** Draw the least number of possible lines going through the rows and columns that have the 0 entries. Let this number of lines be *m*.

**if** *m* = *k* **then**

 The optimal matching is reached and is represented by the corresponding zeros;

**else**

 (Generate additional zeros);

 Find the smallest entry not covered by any line and substract this entry from each row that isn’t crossed out, and then add it to each element that is crossed out twice in the lines;

 Go back to **Step 3**

**end**

**Algorithm 3**: Overall algorithm

**Step 1** Start with the original distance matrix *M* of *n* × *n* elements;

**Step 2** Given the distance matrix *M* of size *k* × *k*, run the Hungarian algorithm on *M* to obtain *σ*;

**Step 3** For this *σ* retrieve the *k*′ permutation cycles and the new intra–cluster–distance matrix *M*′;

**Step 4** Repeat Steps 2 and 3 until *k*′ = *k*.

## 4 Numerical examples

In this section we present a number of numerical illustrations; involving three real data sets, and two simulated data sets. These include both Euclidean and non–Euclidean spaces.

### 4.1 Old Faithful data

This dataset is known in the literature; see e.g. [22]. Some background: Old Faithful is a geyser in Yellowstone National Park. The geyser erupts, which lasts a certain amount of time, and then an interval of quiet until the next eruption. The data consists of consecutive pairs of duration and intervals of eruptions. There are in total 272 data points of bivariate data and we apply the clustering algorithm to these points.

**Fig 2:**
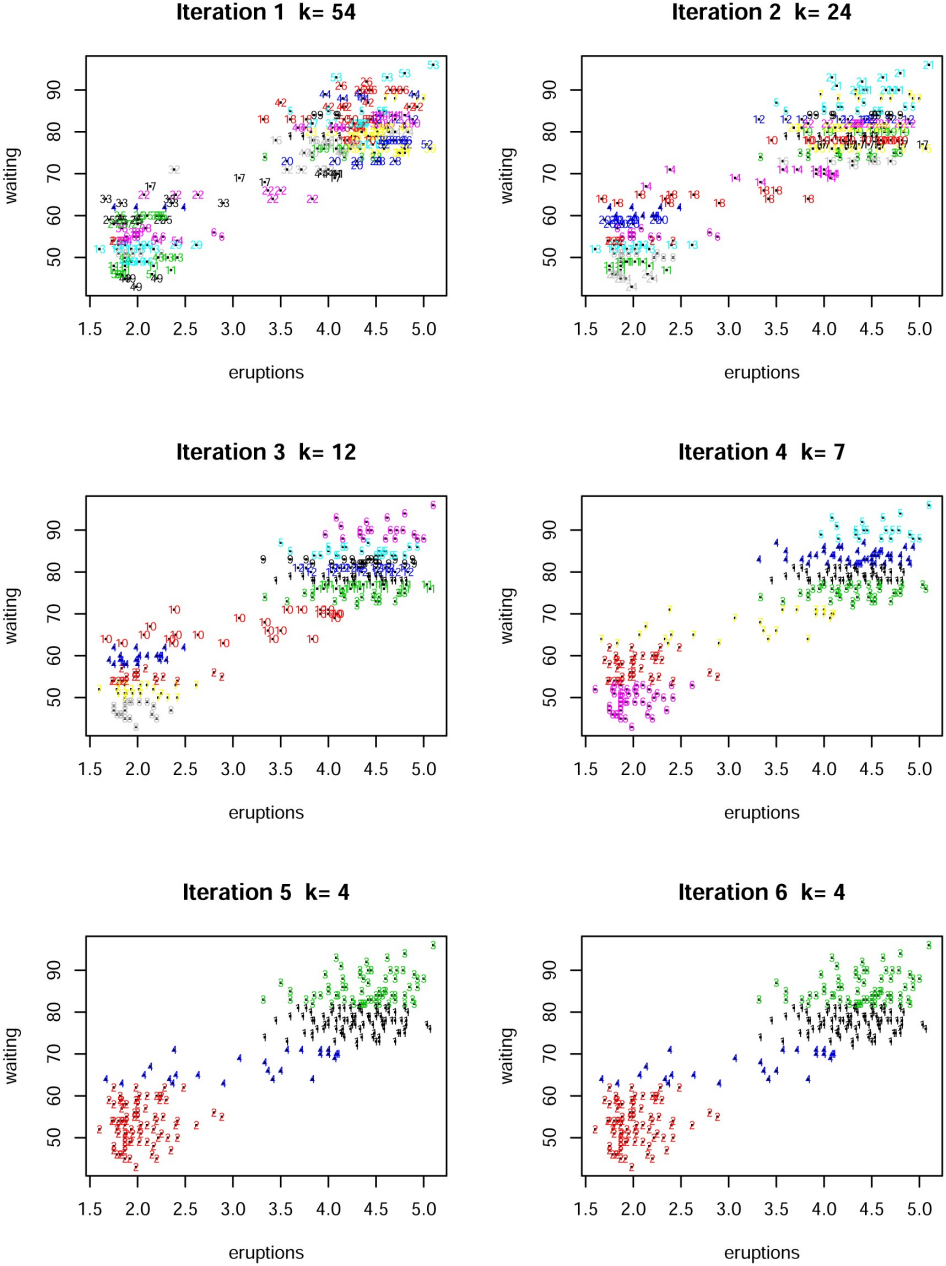
A number of the iterations with current clusters for the Old Faithful data set. The final number of clusters is four.

The algorithm converged after 5 iterations as shown in the Fig 2; note that the plots for iteration 5 and 6 are the same. Hence, the number of clusters chosen by the algorithm is 4.

**Fig 3:**
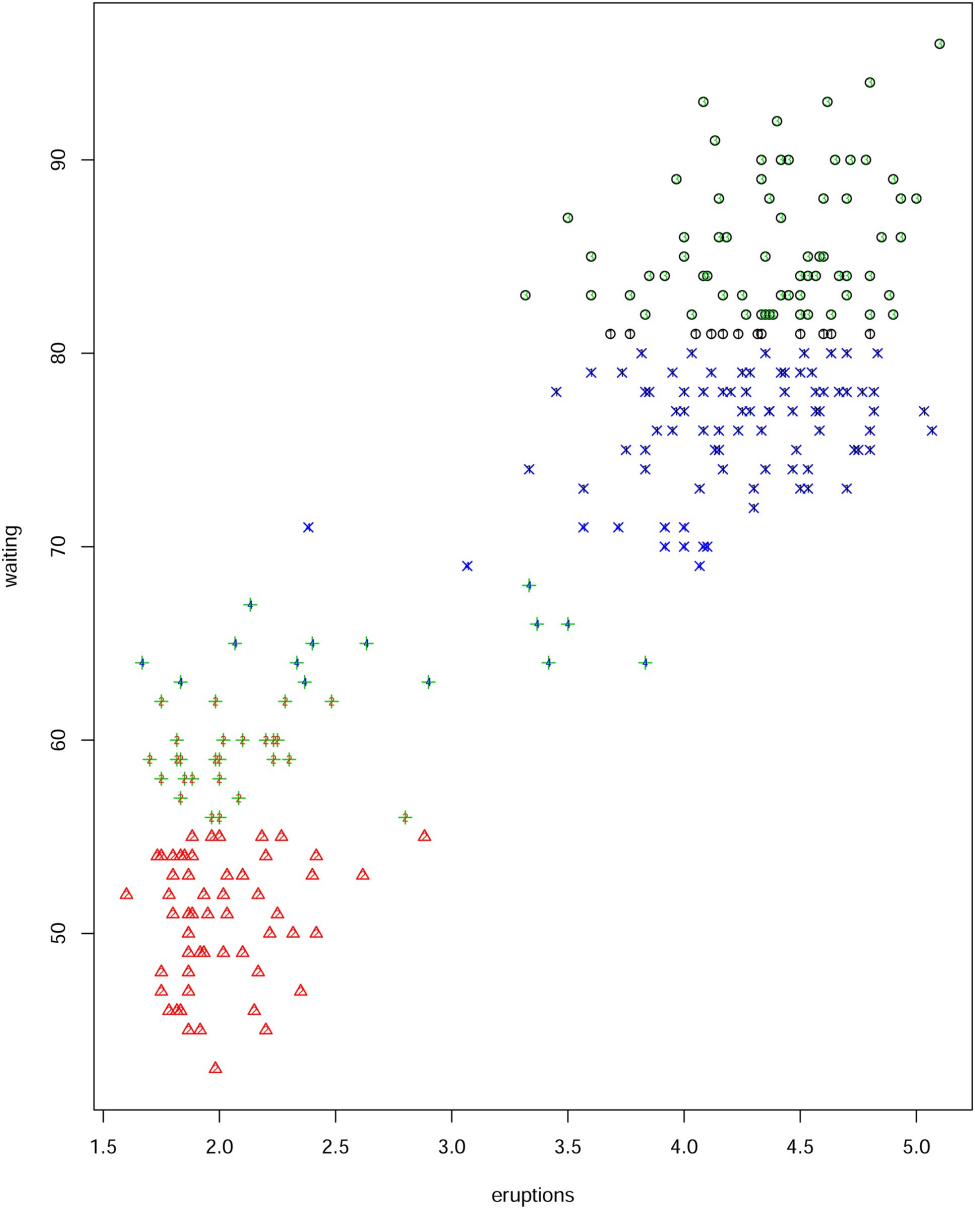
A comparison of the clustering in Fig 2 with the *K*-means output for four clusters.

We also run a *k*–means algorithm in R for four clusters. A visual comparison is given in Fig 3 which confirms that both methods generate essentially similar clusters despite optimizing on different loss functions. Further, [23], with their Bayesian model, achieve a posterior distribution which has a mode at 4 clusters. Hence, our algorithm performs extremely well when and is consistent with alternative approaches described in the literature.

### 4.2 Galaxy data

**Fig 4:**
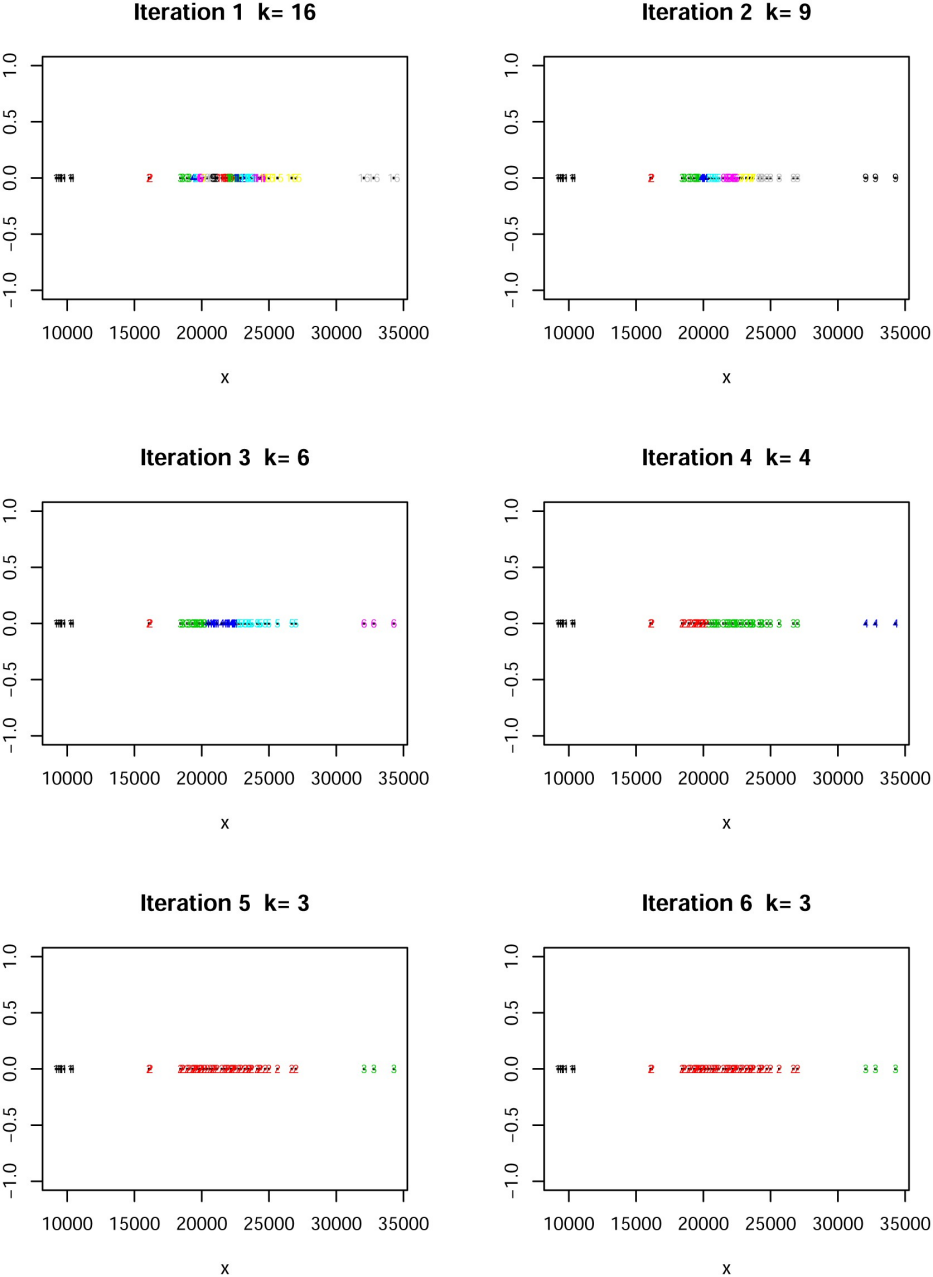
A number of the iterations with current clusters for the Galaxy data set. The final number of clusters is three.

This data set has been extensively studied from a clustering context with a modeling framework using models of the type (3); see, for example, [10]. These models tend to overestimate the number of clusters; see [14]. On the other hand, when the issues with using the mixture model are adequately taken into account, to prevent the overestimation, the number of clusters has been reliably estimated at three; see [24] and [25]. Three is precisely the number of clusters we obtain using our sequential algorithm. The sequence of clusterings is presented in Fig 4.

### 4.3 Simulated data

**Fig 5:**
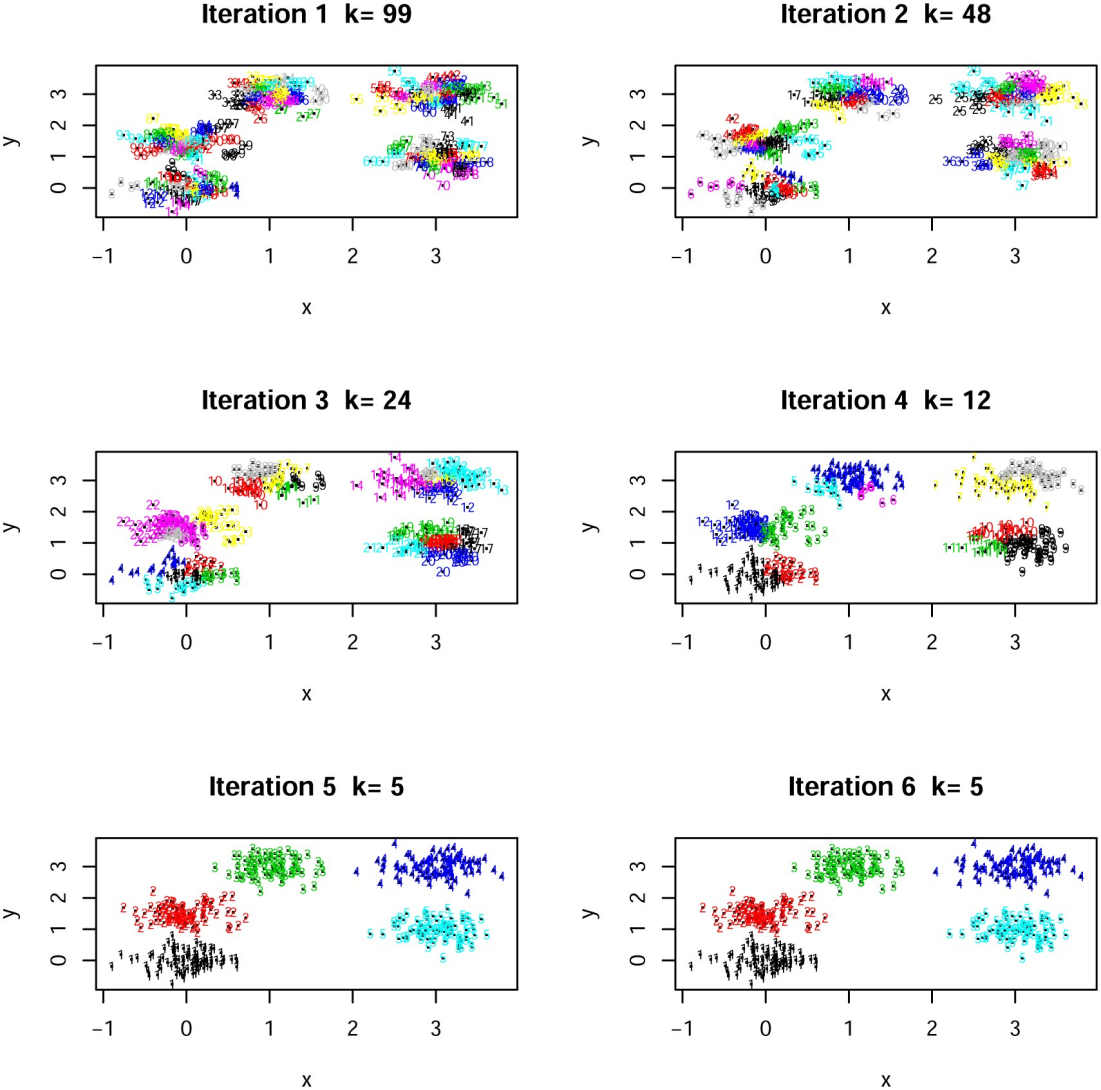
A number of the iterations with current clusters for the simulated data set. The final number of clusters is five—the correct number.

In a first example we simulate 450 random data points around 5 centers in two dimensions with coordinates *C*_1_ = (0, 0), *C*_2_ = (1, 3), *C*_3_ = (3, 3), *C*_4_ = (3, 1), and *C*_5_ = (0, 1.5) and a choice of perturbation standard error 0.3. As seen in Fig 5 our algorithm performs well as it picks the correct number of clusters (five in our example) with visibly correct locations.

**Fig 6:**
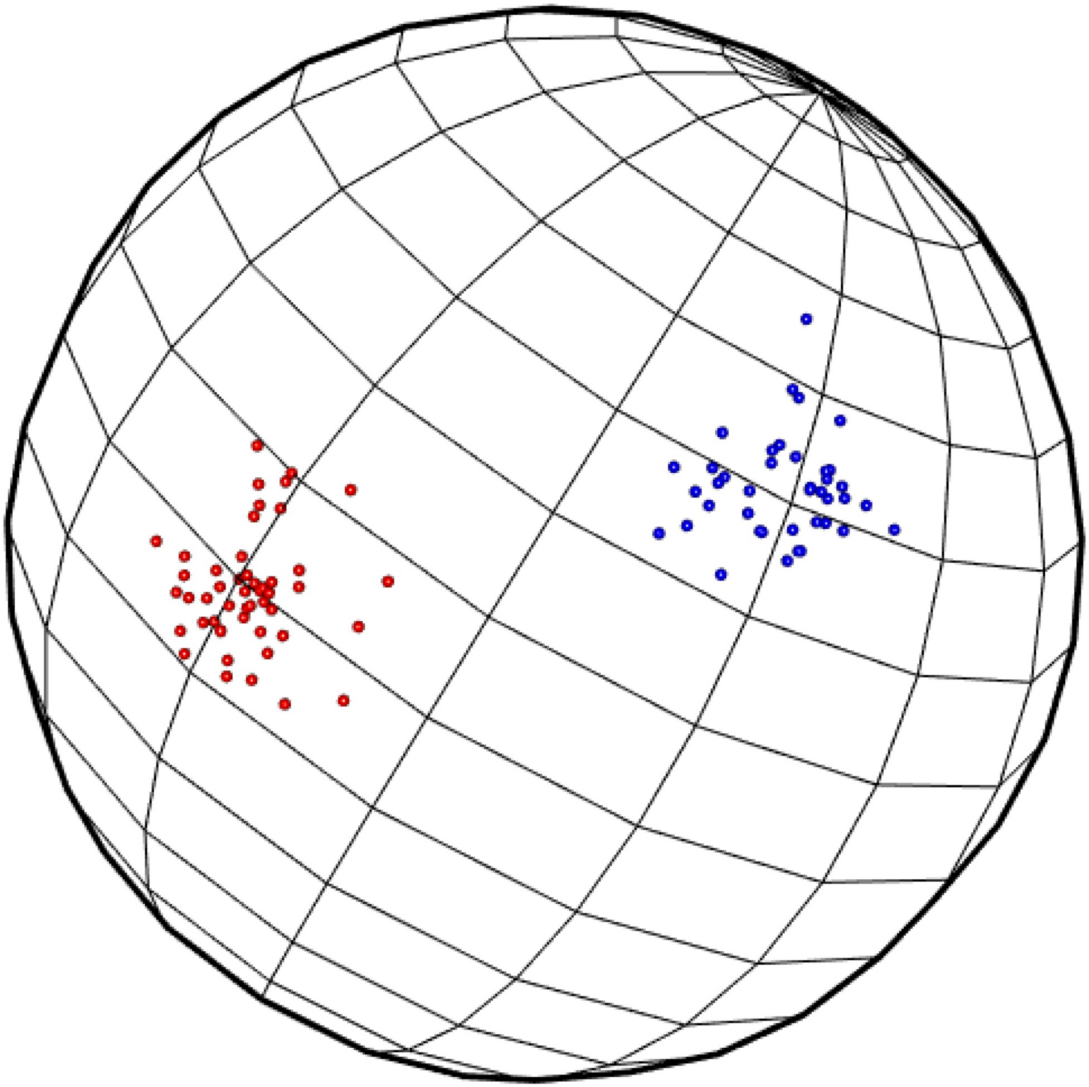
Data set on unit sphere.

For a second example we consider data on a unit sphere. They are projected normal random variables with different means, each mean indicating a different cluster. In Fig 6 the simulated data are shown with one group of size 50 and the other of size 40. The distance used is the arc length and the cluster averages are calculated using the standard mean operation with the co–ordinates on the sphere; i.e. extrinsic means. Starting with 90 clusters, iteration one produces 42 clusters; iteration two produces 20; iteration three 9; iteration four has 5; and the final one has 2 clusters, which separates out the two groups perfectly.

### 4.4 Landmark data

**Fig 7:**
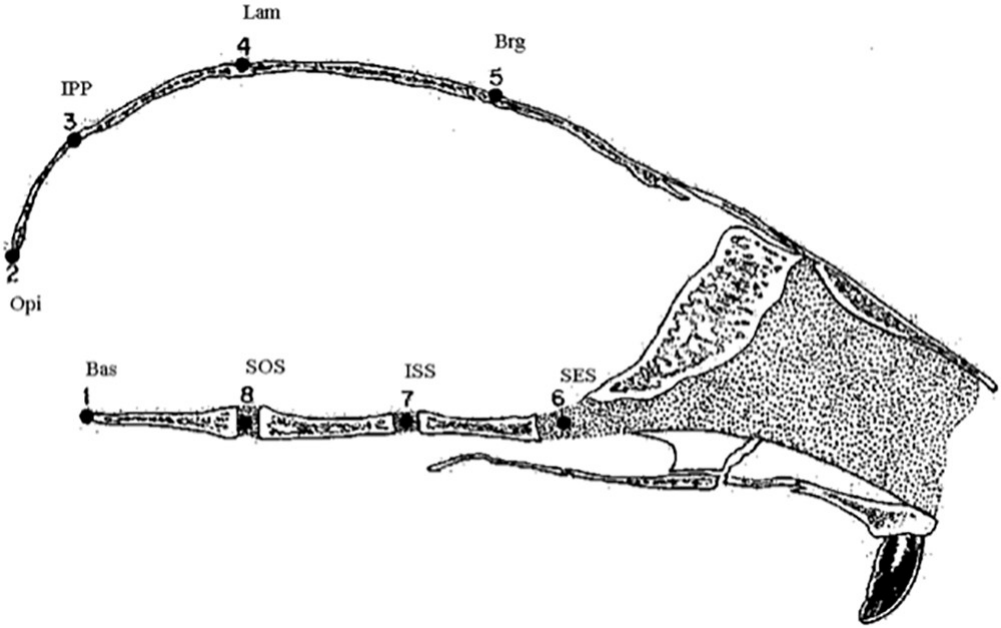
Locations of the eight landmarks.

One of the key advantages of the algorithm is that we rely only on the distance matrix. Such a feature is preferred especially in non–Euclidean spaces. For example, the shape spaces of landmark data are naturally defined as Riemannian manifolds of non negative curvature. Note that the additional requirement in the iterative implementation of our algorithm is that we need to update, at each step, the distance matrix with those of the intra–cluster distances. In this example, we will use the Riemannian shape distance between the corresponding Procrustes means which are a version of the centroid (Fréchet) means for the sample shapes. In the following, consider a classic data set in the shape literature. This is the rats data of [26], which consists of skull observations of 18 individual rats when they are 7, 14, 21, 30, 40, 60, 90 and 150 days old. So in total we have 144 individual skull observations. See Fig 7 for a biological explanation of the 8 landmarks.

**Fig 8:**
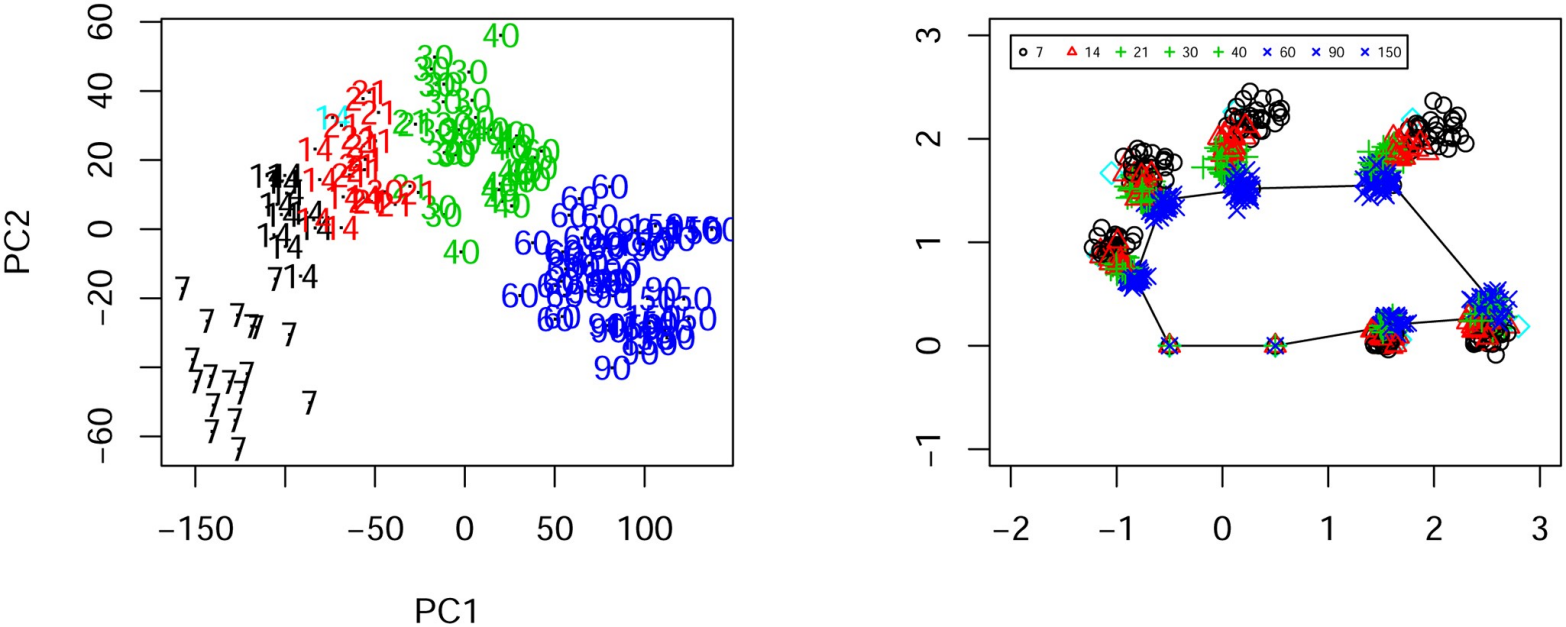
Clustering of the rats data. Left plot: the coordinates of the first two principal components in the tangent space; Right plot: Landmarks represented by symbols and colours based on the clusterings.

We want to explore whether there is any natural clustering the shapes of the skulls depending on the age. Note that in order to extract the shape coordinates one can rescale, relocate and rotate each configuration so that two given landmarks are fixed to two pints. See for example the two fixed landmarks to points (−1/2, 0) and (1/2, 0) in the right figure in Fig 8.

The relevant calculations for obtaining quantities such as the Riemannian shape distances and Procrustean means are carried out by utilising the R-package shapes. Our algorithm for these 144 shapes observed at eight time points, produces five clusters we represent them as color coded in Fig 8.

Since the shape change is more pronounced at the early stages, the shapes observations for ages from 7 to 40 days old, are split into three clusters. However the shape observations for the later stages of 60, 90, and 150 days (blue coloured) are considered as one cluster. This makes sense as the shapes of the skulls changes more at the early growth stages. As it can be seen from the graphical output we could not see any visual evidence of this clustering in the landmark space (right plot in Fig 8). However the plot of the first two principal component scores, in tangent space approximation of the shape space seem to support the clustering choice. This is because due to the nature of the shape metric, such Euclidean (landmark) coordinates cannot immediately display any visible clustering.

### 4.5 Comparison with number of clusters

The *k*–means type algorithms where the number of clusters need to be specified, or hierarchical algorithms which end with a single cluster, require additional procedures to set the number of clusters when unknown. There are a number of techniques; including the most common “elbow” method. This computed the within cluster sum of squares (WSS) as a function of *k* and looks for the integer value for which the subsequent value does not reduce the WSS sufficiently. Other ideas which we will compare with include the “silhouette”, which measures how well each point lies within its cluster, and see [27] for details. Also we consider the “gap” method, which provides the cluster structure most removed from the random uniform distribution; see [9].

These methods for determining the number of clusters uses the function *fviz-nbclust* and can be found in the R package “factoextra”. It computes the appropriate number of clusters for a variety of algorithms, *k*–means, *k*–medoids, and hierarchical *k*–means, though we focus solely on the *k*–means.

**Fig 9:**
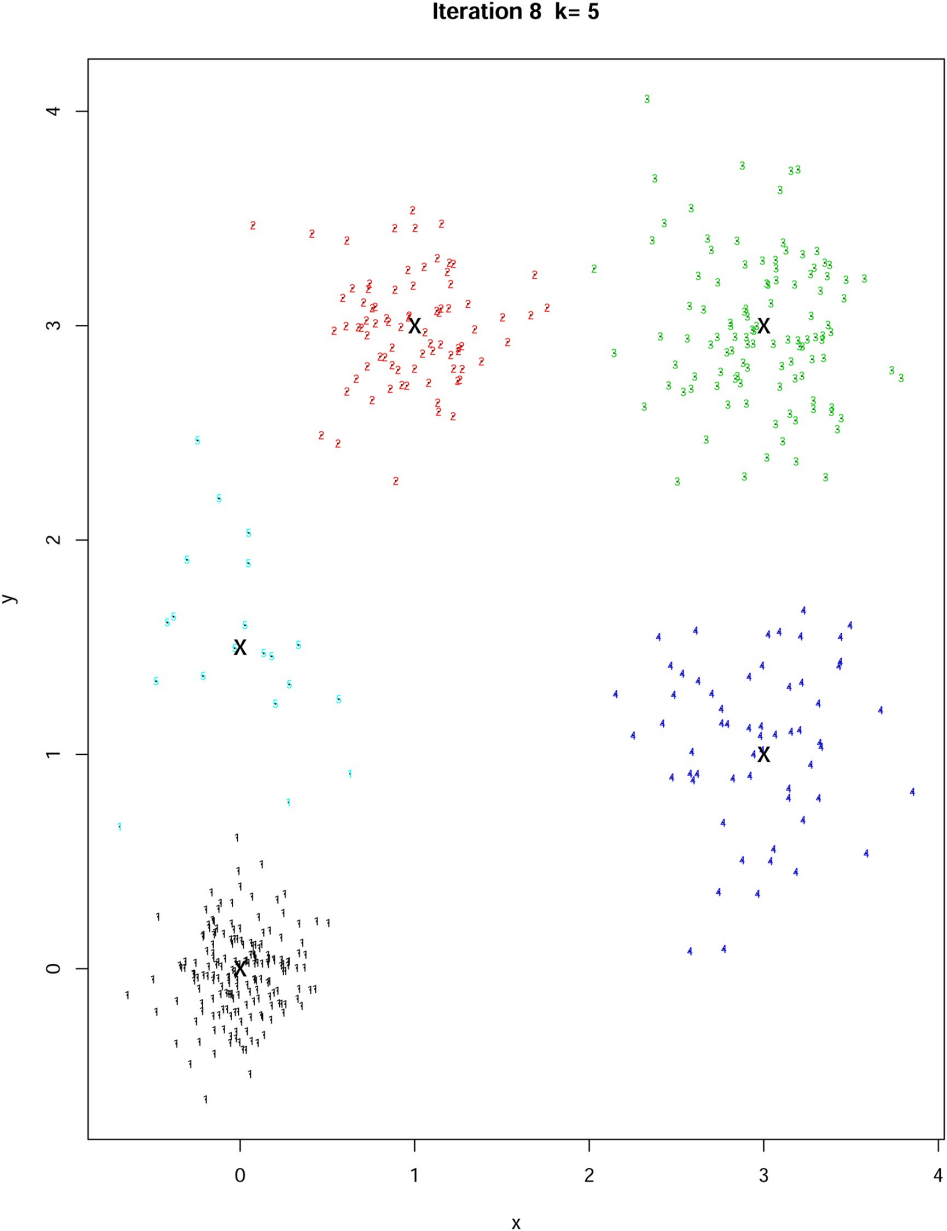
Data set with 5 clusters and *σ*^2^ = 0.2.

The set up for the simulation study involves data sets in 2 dimensions, where the number of clusters is known to be 5. To generate the datasets we fix 5 points, those set in section 4.3, and we generate a number of observations from each of the 5 locations using independent bivariate normal random variables with variance *σ*^2^. We focus in particular on datasets which are biased; i.e. there is a difference in the sizes of clusters as 180, 80, 110, 60, and 20, respectively. See Fig 9 for an illustration of a dataset; the 5 cluster centres are indicated with a cross. The correct number of clusters was obtained in 8 iterations of the Hungarian clustering algorithm.

We kept the same number of elements per cluster and generated the data via 5 normal distributions and changed the variance, labelled as *σ*^2^, of the data for each cluster. So the larger the *σ* is the larger is the cluster overlap. Over multiple runs we take the average number of clusters for each approach, and the average number of clusters is reported in Table 1. That the average is a number is due to the fact that every run, out of 50, always returned the same number of clusters.

**Table 1:** The average number of clusters by after adding noise to the five centres with various *σ*.

*σ*^2^	0.1	0.2	0.3	0.4	0.5
Hungarian	5.	5	5	5	7
Gap	5	5	4	3	2
Silhouette	5	4	4	2	2

We notice that as the perturbation variance *σ* rises, the correct number of clusters is found by the Hungarian clustering algorithm, while the gap and silhouette methods fail, and underestimate the correct number of clusters. For the run times, taking the *σ*^2^ = 0.1 case, the Hungarian algorithm took 0.56 seconds to run 8 iterations starting with 450 observations. The gap method took 1.05 seconds using the clusGap function, the silhouette method took 0.03 seconds using fviz-nbclust.

**Fig 10:**
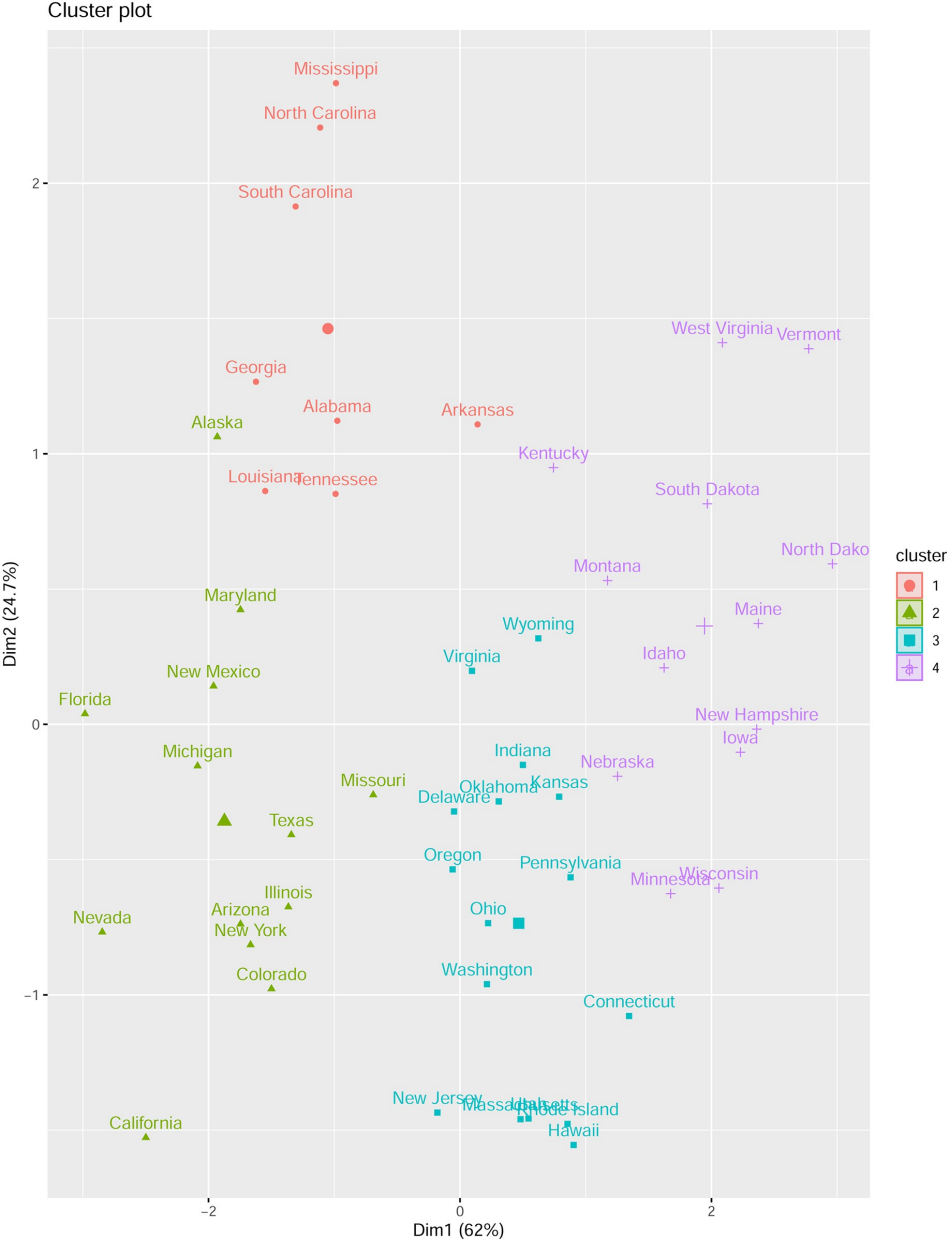
US arrests clusterings from the hierarchical *k*-means algorithm and the gap method.

For a real dataset, we also investigate the number of clusters for the “US arrests” data set, which the above mentioned package use in their documentation. In fact, the silhouette and gap criteria (as well as elbow) find 4 clusters for this data set. See Fig 10.

**Fig 11:**
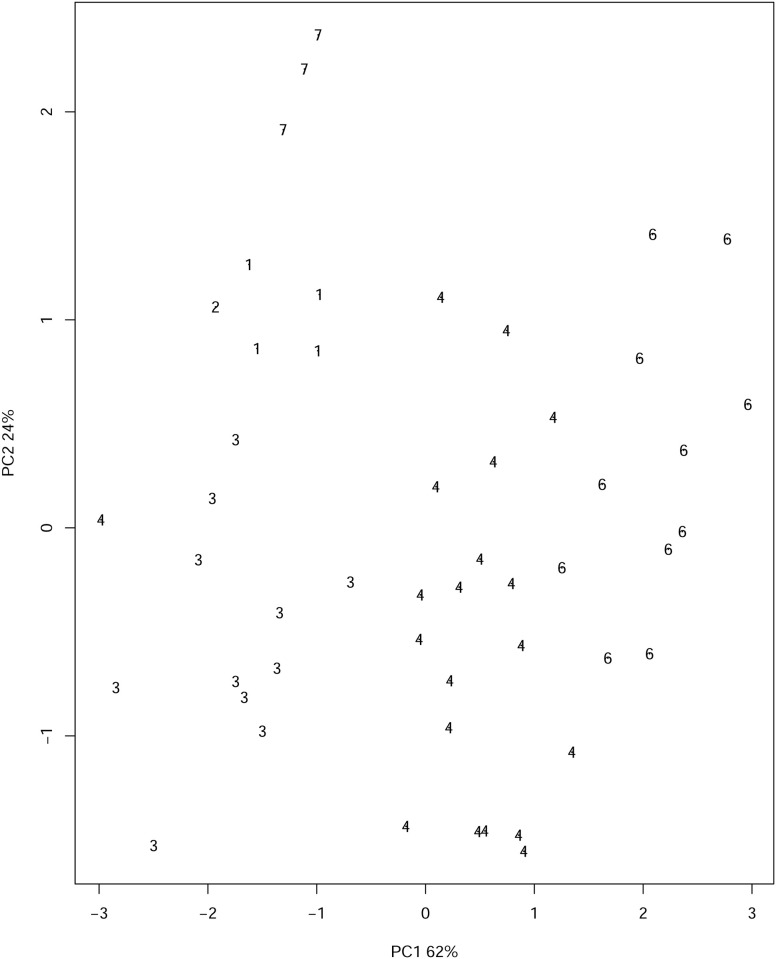
US arrests clustering from the Hungarian algorithm; there are 7 clusters.

**Fig 12:**
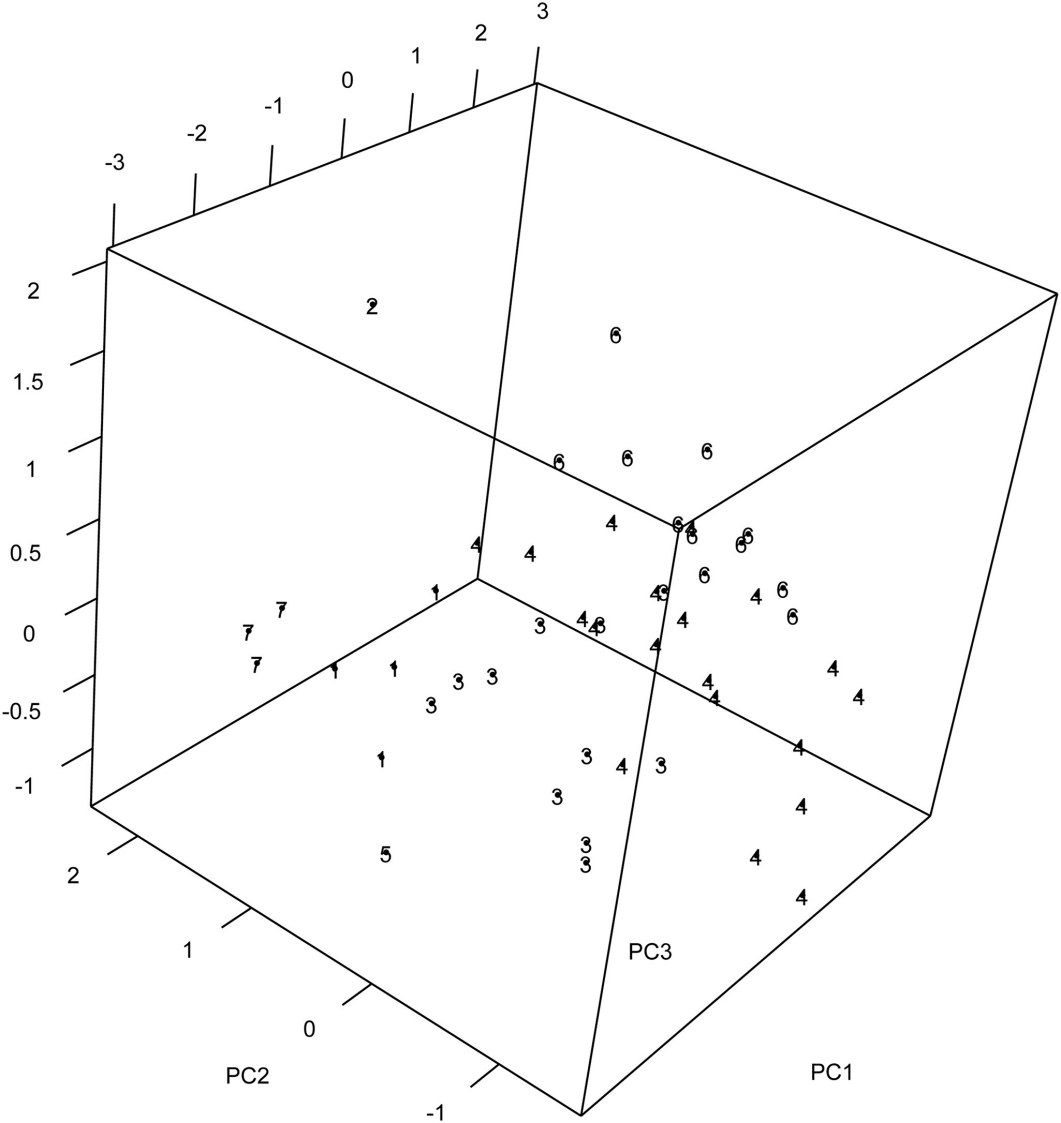
Supporting evidence of 7 clusters for the US arrests data based on view with 3 principal components.

Our Hungarian algorithm, however, suggests 7 clusters; see Fig 11. In light of the simulation study this is not surprising, as we have highlighted the point that these methods seem to underestimate the number of clusters. Our method provides three additional clusters; Florida (cluster number 5), Alaska (cluster number 2) and (Missisipi, South Carolina, North Carolina) (collectively cluster number 7) as in the middle plot. This choice is supported in the the 3–dimensional plot where the third principal component is included; see Fig 12.

## 5 Discussion

At the heart of our clustering approach is the idea of the permutation acting on an objective function over clusters and numbers of clusters. The cyclic groups determine the number and the elements of the clusters. The objective function is evaluated using our value of clusters, which includes one for a single point. Even though it is a hierarchical algorithm, it is quite different to alternatives which do not operate with a specific objective function. The main difference is that our algorithm provides a natural stopping rule; i.e. when the permutation becomes the identity permutation.

The intra–cluster–distance matrix is an important component in our approach. If the data are Euclidean then these distances can be easily calculated as the distance of the corresponding means in the usual sense. However, if the data were in some general non–Euclidean metric space then we replace these distances with those of the corresponding Fréchet means for each cluster. Alternatively, we could use the distance between clusters as the minimal pairwise distance between different clusters, or the maximal pairwise distance, or perhaps more reasonably the average pairwise distance between the clusters. To see this explicitly, if the number of current clusters is *k*, we minimize

$$\sum_{j=1}^{k}\{d\left(S_{j},S_{\sigma (j)}\right)+\eta \;1\left(\sigma \left(j\right)=j\right)\}$$

over all permutations on {1, …, *k*}. We rely on the distance *d* as being the distance between centroids of the clusters (*S*_*j*_). We coud equally consider an alternative when the computing of the centroid is problematic due to a non–Euclidean space. For example, we could use a distance from hierarchical algorithms, such as

$$d\left(S_{j},S_{l}\right)=\min_{i\in S_{j},i^{\prime }\in S_{l}}\left\{d\left(x_{i},x_{i^{\prime }}\right)\right\}.$$

Other popular distances include the avarage distance between the two clusters; i.e.

$$d\left(S_{j},S_{l}\right)=\frac{\sum_{i\in S_{j},\;i^{\prime }\in S_{l}}d\left(x_{i},x_{i^{\prime }}\right)}{|S_{j}\left|\;\right|S_{l}|},$$

and the maximum distance,

$$d\left(S_{j},S_{l}\right)=\max_{i\in S_{j},i^{\prime }\in S_{l}}\left\{d\left(x_{i},x_{i^{\prime }}\right)\right\}.$$

Such algorithms would clearly be accelerating the standard hierarchical clustering algorithm, allowing for more mergers of clusters than one per iteration. We could also consider adapting the *η* to be cluster specifi; i.e. we minimize

$$\sum_{j=1}^{k}\{d\left(S_{j},S_{\sigma (j)}\right)+u\left(S_{j}\right)\;1\left(\sigma \left(j\right)=j\right)\}$$

where *u*(*S*) is given by (4) if |*S*| > 1 and is *η* if |*S*| = 1.

In future work we will look at further examples in non–Euclidean spaces, such as spaces of phylogenetic trees; see [28], for example.

## Supporting information

S1 Datasets (ZIP)
